# Diabetic Myonecrosis of the Axilla: A Novel Case With Severe Clinical Features

**DOI:** 10.7759/cureus.46028

**Published:** 2023-09-26

**Authors:** Kalvin Zee, Michael Poplawski, Jesse Coleman, John Popovich

**Affiliations:** 1 General Surgery, MercyOne Medical Center, Des Moines, USA; 2 General Surgery, Des Moines University, Des Moines, USA; 3 Surgery, MercyOne Medical Center, Des Moines, USA

**Keywords:** septic shock, myonecrosis, diabetic ketoacidosis, diabetes mellitus, diabetic myonecrosis

## Abstract

Diabetic myonecrosis is a rare and poorly understood complication of long-standing, inadequately controlled diabetes mellitus. Theoretical mechanisms contributing to the pathophysiology of diabetic myonecrosis include microvascular complications due to advanced glycation end-products, ischemia-reperfusion injuries, and dysregulated coagulation-fibrinolysis activity. Case reports of diabetic myonecrosis most commonly describe diabetic patients with chronically poor glycemic control who experience isolated swelling and severe pain in a unilateral lower limb with no signs of infection or systemic toxicity. Due to the rarity of this condition, there are currently no treatment guidelines. This case describes a 58-year-old male with a history of uncontrolled diabetes who presented with diabetic ketoacidosis with mixed hypovolemic and septic shock. Diabetic myonecrosis was incidentally discovered in the patient’s right latissimus dorsi with CT imaging and subsequent surgical exploration. Spontaneous diabetic myonecrosis may mimic several other serious conditions and elicit suboptimal management strategies, particularly in the context of atypical presentations.

## Introduction

Diabetic myonecrosis is a rare complication of long-standing, poorly controlled diabetes mellitus. The literature describing this infrequently diagnosed condition largely consists of case reports and case series. Spontaneous skeletal muscle necrosis associated with diabetes mellitus commonly presents with severe pain, swelling, and tenderness in the large muscles of a unilateral lower extremity. This condition may closely mimic a number of serious medical diagnoses, but notably absent are signs of an infectious process, overt coagulopathy, systemic toxicity, or a history of trauma. Currently, diabetic myonecrosis is attributed to a multifactorial combination of microangiopathy, ischemia-reperfusion injury, and dysregulated coagulation-fibrinolysis activity. Case reports often describe diabetic myonecrosis occurring in isolation from other diagnoses or new health concerns. In light of considerable diagnostic difficulty, magnetic resonance imaging and biopsy are frequently described as the gold standard for diagnosis. Currently, there is no standard treatment approach once a diagnosis is established. Treatment approaches commonly include restoring glycemic control, bed rest, non-steroidal anti-inflammatory drug therapy, anticoagulants, and surgical debridement. This case involves a novel distribution of spontaneous skeletal muscle necrosis in the right axilla, in addition to features of a severe concurrent illness.

## Case presentation

The patient is a 58-year-old male with a past medical history of diabetes mellitus with diabetic ketoacidosis, chronic pancreatitis, and heavy substance abuse who presented to the emergency room with acute toxic metabolic encephalopathy. The patient reportedly was not taking his insulin as prescribed and had been feeling weak for three days with nausea and non-bloody emesis. He was subsequently found with an altered mental status and was transported to a regional hospital for a presentation concerning diabetic ketoacidosis.

In the emergency department at the outlying facility, the patient was normotensive but tachycardic, with serum chemistries suggestive of severe metabolic acidosis, diabetic ketoacidosis, and leukocytosis. The patient was immediately started on an insulin infusion, fluid resuscitation, and electrolyte replacements.

**Figure 1:**
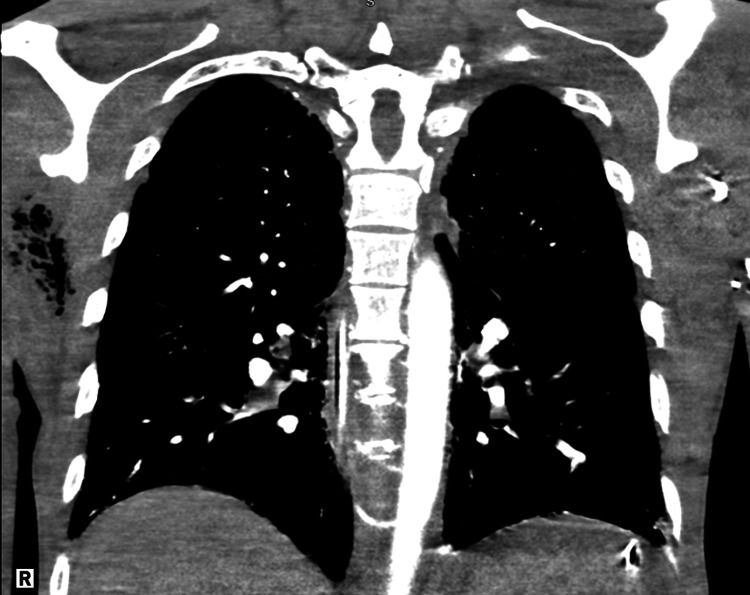
CT angiography chest with extensive soft tissue gas and edema in the right shoulder and axilla

Overnight, the patient became unresponsive, hypotensive, and tachypneic. The patient was placed on mechanical ventilation, norepinephrine, vasopressin, epinephrine, and broad-spectrum antibiotics with vancomycin and piperacillin-tazobactam. A CT angiography scan of the chest revealed extensive soft tissue gas and edema within the right shoulder and axilla (Figure 1). The patient was subsequently transferred to a tertiary care center.

**Figure 2:**
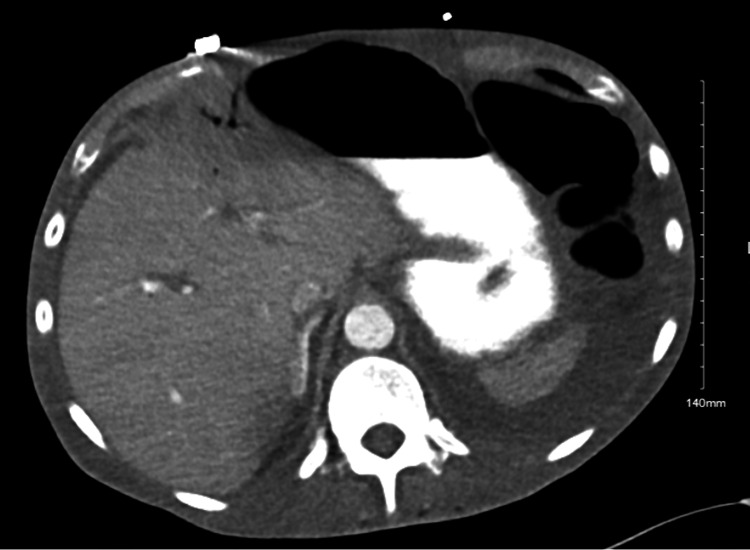
CT abdomen with portal venous gas

**Figure 3:**
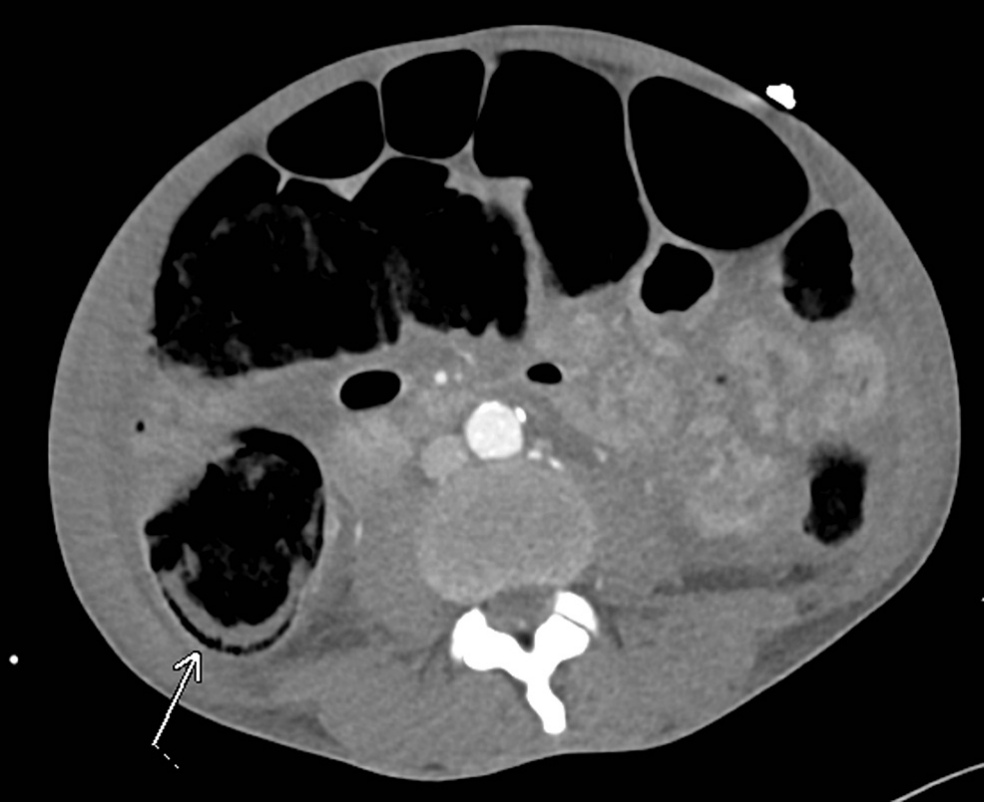
CT abdomen with pneumatosis

Upon arrival, the patient was intubated, sedated, and on norepinephrine, vasopressin, and epinephrine. The patient’s abdomen was found to be distended and rigid. A CT scan of the abdomen and pelvis showed pneumatosis, small free air, and portal venous gas consistent with bowel ischemia (Figures 2-3). At this time, the decision was made to proceed to the operating room.

The patient was brought to the operating room for an exploratory laparotomy. Upon entry to the peritoneum, foul-smelling peritoneal fluid was encountered. A significantly dilated colon from the cecum to the proximal descending colon was discovered with significant stool burden. Fifty centimeters of the terminal ileum, cecum, and right colon appeared gangrenous and were resected en bloc. The superior mesenteric artery was shown to have an appropriate biphasic Doppler signal. The lesser sac was explored and no pathology was found. A temporary abdominal closure was placed and attention was turned to the right axilla.

A 3-centimeter incision along the mid-anterior axillary line of the right axilla was created. Necrosis of the latissimus dorsi was found without fascial necrosis, consistent with diabetic myonecrosis. No purulence was noted, although there was thrombosis of the veins supplying the axillary musculature. At this time, the patient became increasingly unstable with escalating vasopressor requirements; thus, the operation was aborted and the patient was transferred to the ICU. Debridement and biopsies were unable to be completed.

On arrival at the ICU, continuous renal replacement therapy was attempted but aborted due to the patient’s worsening hypotension. The patient was on maximum doses of norepinephrine, vasopressin, epinephrine, and angiotensin. Dobutamine was started. Despite the intensive support measures, the patient’s condition continued to deteriorate. The family was contacted, and the patient was placed on comfort measures. He expired soon after with family at the bedside.

## Discussion

Diabetic myonecrosis is a rare complication of type 1 and type 2 diabetes mellitus. Risk factors include extended duration of diabetes and uncontrolled hyperglycemia. This condition is poorly studied and likely underreported. Since the first description of diabetic myonecrosis in 1965, only 200 cases have been described in the literature in the preceding 50 years [1,2]. Notably, spontaneous diabetic muscle infarction is distinct from other etiologies of myonecrosis, such as necrotizing soft tissue infection, arterial thrombosis, and pressure necrosis. The pathophysiology of this condition is not well elucidated, but proposed mechanisms for skeletal muscle involvement include microangiopathy, ischemia-reperfusion injury, and dysregulated coagulation-fibrinolysis activity [3]. Supportive evidence of this theoretical basis is the considerable comorbidity of end-stage complications of diabetes, including neuropathy, retinopathy, and nephropathy [4]. Common features of diabetic myonecrosis are localized severe pain, swelling, and tenderness most commonly in the unilateral thigh or calf musculature. Before 2013, a case of upper limb diabetic myonecrosis had yet to be reported [5]. Clinical signs and laboratory values in these cases are often nonspecific, leading to an extensive differential diagnosis [6]. Due to the infrequent incidence of the condition and limited data from diabetic myonecrosis literature, the treatment approach is not standardized. Mainstays of treatment include conservative measures such as bed rest, non-steroidal anti-inflammatories, and restoring glycemic control [2]. Other treatment approaches include antiplatelet therapy and surgical debridement [7]. Notably, nonsurgical management of diabetic myonecrosis is associated with lower recurrence and mortality rates in the subsequent two years [8]. In 2005, a review of the literature yielded 49 similar patients diagnosed with diabetic myonecrosis. Average recovery times from treatment onset were 5.5 weeks for patients treated with antiplatelet and/or anti-inflammatory drugs, eight weeks for bed rest with analgesia, and 13 weeks after surgical debridement. The surgical treatment group had the highest associated rates of recurrence and mortality among the treatment groups [8].

In this clinical case, the additional factor of muscle infarction may have exacerbated the clinical status of the patient, contributing to rapid deterioration. There was considerable diagnostic uncertainty due to the obtunded condition of the patient upon presentation. Clinical staff was not able to rely on typical features of the history such as focal pain and tumoriform swelling in the absence of trauma or the degree of diabetes medication compliance. Upon incidental discovery of soft tissue emphysema on CT angiography of the chest, the decision was made to surgically explore the right axilla. Considering the patient’s clinical picture, we maintained a high level of suspicion for necrotizing soft tissue infection. Surgical exploration revealed partial necrosis of the right latissimus dorsi without fascial necrosis or evidence of infection. Thrombosis of veins supplying axillary musculature was noted at the time, but there were no obvious signs of purulence, exudate, or foul smell. Immediately following this discovery, the patient’s worsening clinical status led to the discontinuation of surgical exploration, and tissue biopsy was deferred. The patient was then returned to the ICU, where he expired shortly thereafter.

This case presents several features that may be newly attributable to diabetic myonecrosis. In contrast to previously described cases, diabetic myonecrosis in the context of distributive shock and bowel necrosis is uncommon, lacking description in published literature. Furthermore, the involvement of the axilla and trunk via the latissimus dorsi has not been referenced in the literature to the best of our knowledge. Unfortunately, due to the overall clinical picture, surgical exploration of the affected area was pursued. It is possible that this exploration may have inadvertently caused the extension of muscle necrosis. The evolving literature on diabetic myonecrosis suggests that surgical interventions are associated with longer recovery times and higher rates of recurrence.

In discussing this rare and challenging condition, we would like to put forth a new theoretical mechanism contributing to the patient’s development of diabetic myonecrosis. A possible contributing factor to the case may have been the patient’s recent use of methamphetamine, as reported on the urine drug screen. The timing of substance use coincides with the patient’s worsening confusion, three days prior to presentation. Methamphetamines are a sympathomimetic substance inducing neurotransmitter and catecholamine release, leading to vasoconstriction and possible tissue ischemia. After methamphetamine is metabolized by cytochrome P450 isozyme CYP2D6 into active metabolites, they are subsequently excreted into urine. Given this patient’s status of septic shock requiring vasopressors, the paucity of renal perfusion may have limited the patient's ability to excrete metabolites, perhaps prolonging their vasoactive effects and contributing to myonecrosis [9].

Initiation of vasopressor therapy with subsequent increase in peripheral perfusion may have contributed to an ischemia-reperfusion injury, augmenting necrosis of the latissimus dorsi muscle. Reperfusion of tissues after ischemia is understood to release damaging oxygen free radicals, cause impairment of endothelial cell-dependent arteriolar dilation, and promote leukocyte plugging of capillaries [10]. As previously suggested in the literature, this mechanism is a plausible explanation for spontaneous focal muscle necrosis. The precise reason for the involvement of right axillary and shoulder musculature in this case is not clear. Additional research is needed to further understand this rare condition.

## Conclusions

Spontaneous diabetic myonecrosis is a rare complication of diabetes mellitus that is poorly understood. Patients typically present with abrupt, focal swelling of musculature that may resemble necrotizing soft tissue infection or vascular thrombosis. Delays in diagnosis and optimal management may occur due to the rarity of the disease. The pathophysiology of this condition is currently considered to be multifactorial, including microangiopathy, ischemia-reperfusion injury, and dysregulated coagulation-fibrinolysis activity. In the unstable patient, surgical debridement may precipitate clinical decline rather than temporizing the necrotic process. Further descriptions of diabetic myonecrosis in this challenging context will enable earlier recognition and optimal medical management.
